# Genetic Diversity and Selection Signal Analysis of Xinjiang Black Pig Based on Whole Genome Resequencing

**DOI:** 10.3390/genes17030293

**Published:** 2026-02-28

**Authors:** Mingming Tian, Yun Feng, Haitao Wang, Qiang Wang, Jingyang Dong, Haichao Zhao, Fahui Yang, Mengxun Li, Guang Pu, Xinyin Zhang, Dan Wang, Guang Li, Hongwei Chen, Tao Huang

**Affiliations:** 1 College of Animal Science and Technology, Shihezi University, Shihezi 832000, China; 20232113001@stu.shzu.edu.cn (M.T.); 18785778959@163.com (Y.F.); 17513767969@163.com (H.W.); 20232013001@stu.shzu.edu.cn (Q.W.); 18899590672@163.com (J.D.); z2571133122@163.com (H.Z.); 15139380816@163.com (F.Y.); mxli98@shzu.edu.cn (M.L.);; 2 The Eighth Division Livestock and Fisheries Development Center, Shihezi 832000, China15299963569@163.com (D.W.); lg405950599@163.com (G.L.); 3 Shihezi Ruikang Breeding Professional Cooperative, Shihezi 832000, China; 4 Xinjiang Pig Breeding Engineering and Technology Research Center, Changji 831100, China

**Keywords:** Xinjiang Black pig, whole genome resequencing, population structure, inbreeding coefficient, selection signal

## Abstract

**Background:** The Xinjiang Black pig is an excellent breed developed by the Xinjiang Production and Construction Corps in the 1990s; however, it has been endangered by the impact of commercial breeds. **Methods:** Whole genomes of 224 individuals from the Xinjiang Black pig conservation population were resequenced. **Results:** Genetic structure and diversity analyses revealed that Xinjiang Black pigs underwent severe inbreeding and were genetically closely linked to Landrace pigs. The genetic diversity of the F_2_ generation was well preserved in the existing breeding scheme. A total of 686 significant selection regions and 406 candidate genes were identified using *F*_ST_ and θπ complementary methods, with Xinjiang Black pigs, Min pigs, and Laiwu pigs as ancestral populations, and F_2_. Based on Gene Ontology, Kyoto Encyclopedia of Genes and Genomes, and quantitative trait loci annotations, potential germplasm candidate genes were identified. Among these, *SOX5*, *HMG20A*, and *NEDD4* are associated with fat deposition; *SPRY1*, *MNS1*, *DMXL2*, and *ALB* are closely associated with male reproductive ability; *ARPP19* and *TLN2* are strongly associated with oestrous cycle regulation and oocyte maturation; and *SLC4A4* and *SLC12A1* are extremely important for osmotic regulation and foetal survival. **Conclusions:** These findings deepen our understanding of the genetic mechanisms of artificial selection in Xinjiang Black pigs and provide a theoretical basis for subsequent breeding and genetic research on this breed.

## 1. Introduction

The social and economic development of humans is profoundly influenced by pigs, which are one of the most widely distributed and adaptable domestic animals worldwide [1,2,3]. At least 235 breeds have been documented across diverse agroecosystems in Asia (https://www.fao.org/dad-is (accessed on 11 November 2024)). According to the National Livestock and Poultry Genetic Resources List (2024 Edition, https://www.nahs.org.cn/ (accessed on 4 March 2025)), 89 local pig breeds can be found in China. Chinese domestic pigs are adaptable, highly fecund, and produce high-quality meat, while possessing a vital gene pool that can be used for pig breeding worldwide [4]. However, 76 domestic pig breeds are distributed across backward areas; owing to the introduction of non-native varieties, nearly one-third of these pig breeds are endangered, and there are extremely few local breed resources that are truly developed and utilised. In this regard, the Chinese government implemented a full-chain support policy for developing native pig breeds. Market data confirm the growth trend in high-grade pork consumption; in 2024, the number of black pigs sold in China reached 21.8 million, up from 19.3 million in 2023.

During the 1970s and 1980s, the rising demand for pork in China prompted crossbreeding and the improvement of indigenous pig breeds through the introduction of foreign varieties. This process has led to the development of numerous dual-purpose breeds optimised for both meat and fat production, including Harbin White, Shanghai White, Beijing Black, and New Golden pigs. Xinjiang Black (XH) pigs are crossbred with pig breeds from the northwest, such as Bamei pigs, and foreign commercial breeds, including Yorkshire, Soviet White, Berkshire, and Landrace (LD), to facilitate crossbreeding and genetic improvement. Consequently, numerous black pig variants preferred by the local population have emerged in the swine population. These black pigs serve as foundational stocks for further selective breeding programs. In 1992, this breed was formally recognised in the publication ‘Swine Breeding in China’.

The XH pig population has been affected by foreign commercial breeds, which have brought it to the brink of extinction, and is facing a reduced population size and severe inbreeding depression. Based on previous germplasm resource screening, our group implemented emergency conservation measures for the endangered XH pig breed and introduced two Chinese native black pig breeds from northern China—the Laiwu pig (LW) from Dongying, Shandong, and the Min pig (MZ) from Lanxi County, Heilongjiang—to enhance its genetic diversity. Furthermore, the incorporation of genetic material from multiple breeds during breeding results in incomplete pedigree records. In this study, whole-genome resequencing was performed on the current XH pig conservation population to genetically rectify pedigree information, assess the effectiveness of conservation efforts, and provide informed guidance for future breeding preservation, development, and utilisation strategies.

## 2. Materials and Methods

### 2.1. Sample Collection and DNA Extraction

A total of 224 samples were collected from the pig farms of the Ruikang Breeding Cooperative in Shihezi. The parental generation comprised 6 XH pigs, 22 MZ pigs, and 9 LW pigs. The F1-generation pigs included 33 Laimin (LM), 8 Laixin (LX), and 53 Minxin (MX) pigs, which were obtained by crossing Laiwu × Min, Laiwu × Xinjiang Black, and Min × Xinjiang Black pigs, respectively. The F_2_ generation included 81 pigs, primarily derived from crosses between MX and LX. In addition, 12 Landrace pigs (LD) were introduced as an outgroup population, sourced from Ruoqiang County, Xinjiang. (Supplementary Table S1). Genomic DNA from each ear tissue sample was extracted using a Tiangen DNA Extraction Kit (Tiangen Biotechnology Co., Ltd., Beijing, China).

### 2.2. Sequencing Data Quality Control

In this study, a DNA sequencing library comprising 224 samples was prepared and sequenced on the DNBSEQ-T7 high-throughput sequencing platform (Gezhiqiyin Biotechnology (Jiaxing) Co., Ltd., Jiaxing, China) using paired-end 150 bp (PE150) reads. The cohort comprised 20 high-depth (20×) and 204 low-depth (2×) samples (Supplementary Table S1). The high-depth samples comprised parental individuals with clear pedigree records and offspring demonstrating excellent performance. High-depth samples (20 samples): The HaplotypeCaller module of GATK (v.4.2.6.1) [5] was used to perform individual variant calling in Genomic Variant Call Format (VCF) mode, followed by joint genotyping to construct a high-precision reference haplotype panel. Low-depth samples (204 samples): Genotype imputation was performed using Beagle (v.5.4), combined with the self-built reference panel to improve genotypic accuracy and single-nucleotide polymorphism (SNP) density (Supplementary Table S1).

The original WGS data were merged and quality controlled using fastp (v0.23.4; https://github.com/OpenGene/fastp (accessed on 28 May 2025)). The tool automatically identifies and cuts the splice sequence, rejects reads with too many low-quality bases, unknown bases, or more than 10% unknown bases, and trims and filters the reads to generate high-quality clean reads and quality control reports in HTML/JSON format.

The quality control clean reads were compared using the Burrows–Wheeler Aligner (BWA, v.0.7.18) to the porcine reference genome Sus scrofa 11.1 [6,7]. The comparison results were converted to the BAM format and sorted based on genomic coordinates using SAMtools (v.1.10) [8]. Subsequently, the PCR repeat reads were removed using the MarkDuplicates tool in GATK (v.4.2.6.1).

All sex chromosomes and ‘NW’ chromosomes were removed from the VCF file, leaving only 18 autosomes for subsequent analysis. Subsequently, VCFTOOLS (v.0.1.17) [9] was used for SNP quality control. The parameters were set to ‘- minDP 4 - max-missing 0.5 - minQ 30 - maf 0.05 - minGQ 10’, implying that only loci with sequencing depth ≥ 4, deletion rate ≤0.5, minor allele frequency ≥0.05, and detection quality ≥ 30 were retained. SnpEff (v.5.4) was used to annotate the resulting VCF file [10].

### 2.3. Analysis of Population Genetic Diversity

VCFTOOLS (v.0.1.17) was used to calculate the expected heterozygosity (HE), observed heterozygosity (HO), nucleotide diversity (π), and Minor Allele Frequency (MAF) according to the sample information—visualisation with the R language.

### 2.4. Population Structure Analyses

To comprehensively analyse the structure of the XH pig conservation population, PLINK (v.1.90) [11] was used for quality control of the filtered VCF files, and the parameters were set to ‘- geno 0.05- maf 0.05 - hwe 1 × 10^−6^ - indep-pairwise 200 kb 200 0.5 - allow-extra-chr’. This procedure yielded 18,680,617 SNPs for further analysis, focusing exclusively on 18 autosomes.

VCF2Dis (v.1.47) was used to calculate the genetic distance matrix between individuals, and a tree was constructed using iTOL (v.7.4.2) [12,13]. To reveal the relationships among different populations of XH pigs, a principal component analysis (PCA) was performed using PLINK (v.1.90) and visualised using RStudio (v.4.5.1). Population structure was inferred using ADMIXTURE (v.1.3.0) [14] with default settings. The optimal ancestral cluster number, K, was estimated using a five-fold cross-validation method. The ancestral pedigree coefficient K was tested in the range of 2–8, and the ancestral genealogy map of the tested varieties was drawn using the R package ‘pophelper’ [15].

### 2.5. Kinship and LD Analysis

PLINK (v.2.00) [16] was used to compute the KING matrix, thereby facilitating the generation of a comprehensive Identity-by-Descent (IBD) table. Kinship relationships were assessed using the KING values, and pedigree adjustments were made in conjunction with genealogical records. The PopLDdecay (v.3.41) software program with default parameters was used to calculate the linkage imbalance values for all populations based on the square correlation (r^2^) between SNPs [17].

### 2.6. Selection Signature Detection of the XH Pigs

Two genomic selection signature detection methods were adopted, including the population differentiation coefficient (*F*_ST_) and the polymorphism level statistic (π ratio). The *F*_ST_ values and π values were calculated separately using VCFTOOLS (v.0.1.17). The genome window and step sizes were set to 50 and 25 kb, respectively, and the first 5% of extreme overlap between the two methods was used as a potential candidate region. The genomes of XH, MZ, and LW pigs were selected as ancestral reference populations to identify selected regions in the F_2_ generation. The corresponding gene annotation files were downloaded from the Ensembl gene interpretation database (https://ftp.ensembl.org/pub/release-115/gtf/sus_scrofa/ (accessed on 23 September 2025)), and the selected regions were annotated using BEDTools (v.2.31.1) [18].

### 2.7. Genome Annotation and Enrichment Analyses

To further analyse the potential biological functions of genes in the candidate region, Gene Ontology (GO) functional annotation and Kyoto Encyclopedia of Genes and Genomes (KEGG) pathway enrichment analysis were performed using R packages, including BioMart (v.2.64.0) [19] and clusterProfiler (v.4.16.0) [20]. Finally, published pig quantitative trait loci (QTLs) were downloaded from the porcine QTL database (PigQTLdb) [21] (https://www.animalgenome.org/cgi-bin/QTLdb/SS/index (accessed on 26 September 2025)) to identify QTLs that overlapped with the candidate regions in this study.

## 3. Results

### 3.1. Detection and Distribution of SNPs

**Figure 1:**
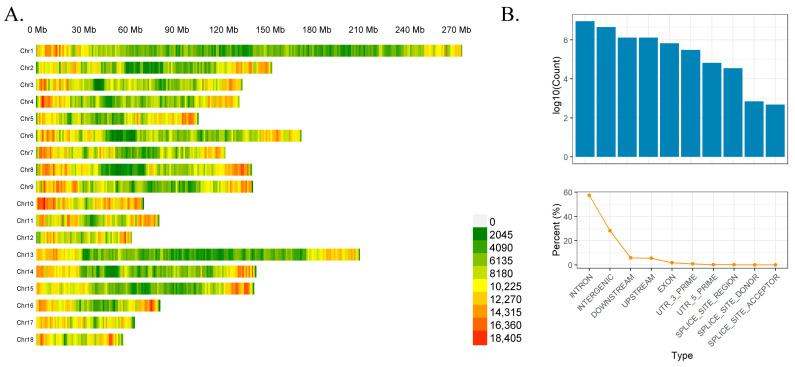
(**A**) Single-nucleotide polymorphism (SNP) distribution map; and (**B**) number of SNPs (**top**) and discovery rate (**bottom**) for different annotated regions.

To investigate the genetic variation characteristics of the Xinjiang Black pig conservation population, whole-genome resequencing was performed on 224 individuals (Supplementary Table S1) using the DNBSEQ-T7 platform. Among these, 20 samples (parents with clear pedigree records and excellent offspring) were subjected to high-depth resequencing at 20× coverage, whereas the remaining 204 were subjected to low-depth sequencing at 2× coverage. Approximately 2270.52 G bases of raw data and 2264.5 G bases of filtered, clean data were produced during this sequencing, with an average of 10,426.065 and 10,398.422 M bases per sample, respectively. To assess the reliability of genotype calls from low-coverage sequencing, we downsampled high-coverage (~20×) whole-genome data to 1× and compared imputed genotypes against the original high-coverage calls. Concordance across 40.9 million autosomal SNPs ranged from 87.8% to 89.9%, demonstrating that low-coverage sequencing combined with imputation yields highly accurate genotypes in this population (Supplementary Table S2). After strict quality control and filtering of the packed SNPs, 18,680,617 SNPs were retained, and the filtered SNPs were annotated and counted using SnpEff (v.5.4). A considerably high number of SNPs, i.e., 20,323,497 (57.416%) and 9,974,382 (28.179%), were located in the intron and intergenic regions, respectively. Only 655,089 (1.851%) SNPs were present in the exon region, including 78,585 (0.222%) non-synonymous and 169,227 (0.478%) synonymous SNPs (Figure 1B, Supplementary Table S3). The number and density distributions of SNPs in different autosomes are shown in Figure 1. The number of SNPs per chromosome was positively correlated with total length (*r* = 0.78). As shown in Figure 1, chromosome 1 exhibited the highest number of SNPs at 1,802,035, whereas chromosome 18 exhibited the lowest number of SNPs among the autosomes at 558,859. SNPs were uniformly distributed on chromosomes, with the highest distribution density of SNPs on chromosome 10 being 11,905.99/Mb, whereas the lowest density on chromosome 1 was 6568.85/Mb. We further analysed the physical location distribution of the SNPs on each chromosome using a 1.0 Mb non-overlapping window, with different colours representing different numbers (Figure 1A, Supplementary Table S4). Overall, the distribution of these SNPs suggests that they are uniformly distributed across each chromosome.

### 3.2. Genetic Diversity of Black Pigs in Xinjiang

**Figure 2:**
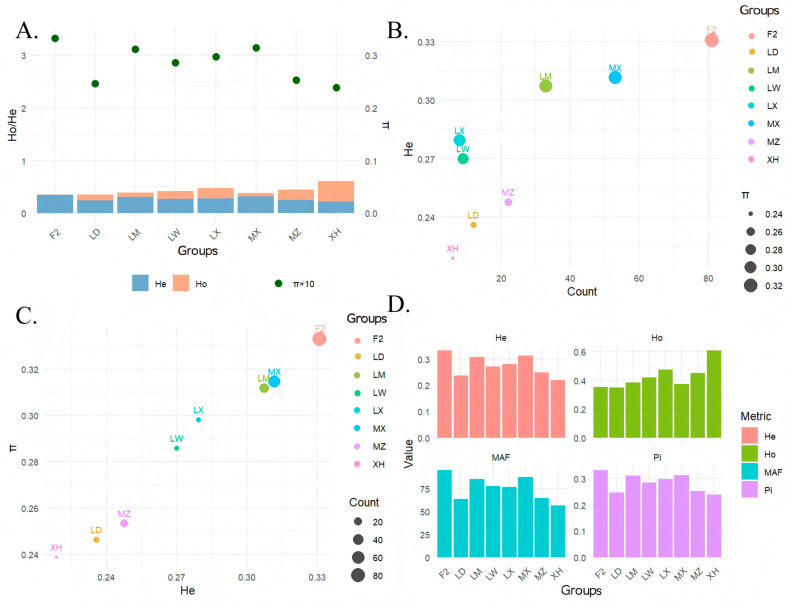
Genetic diversity of the Xinjiang Black (XH) pig conservation population. (**A**) Comparative analysis of heterozygosity and nucleotide diversity across groups; (**B**) Relationship between sample size and genetic diversity; (**C**) Relationship between expected heterozygosity and nucleotide diversity; and (**D**) Indicators of genetic diversity across groups.

The expected heterozygosity (He) and observed heterozygosity (Ho) of each breed were calculated (Figure 2A, Supplementary Table S5), and it can be seen that the observed heterozygosity and the lowest expected heterozygosity (He: 0.218773, Ho: 0.605804) of XH pig populations were calculated, indicating that there was intense inbreeding between XH pig populations, and the potential genetic diversity was extremely low. He and Ho were the closest (He: 0.330701, Ho: 0.353147) in the F_2_ generation, indicating that genetic diversity in the F_2_ generation is effectively preserved (Figure 2A).

The relationship between He, sample number, and nucleotide diversity π was explored, and a positive correlation was observed between He and π, although they did not show an apparent increase or decrease with the number of samples (Figure 2B,C). For example, when the number of samples is 6, He = 0.21877, and when the number of samples is 81, He = 0.330701, the fluctuations in He across different sample sizes are relatively random. In addition, there is no simple linear relationship between π and the number of samples. For instance, when the number of samples is 12, π = 0.246151, and when the number of samples is 53, π = 0.314649. It can be found that when the He value is high, the π value is often relatively high. Conversely, when the He value is low, the π value tends to be lower. For example, when the number of samples is 12, He = 0.235895 and π = 0.246151, and when the number of samples is 22, He = 0.247643 and π = 0.253402, which indicates a positive correlation between the two.

Furthermore, we calculated the SNP ratio for minor allele frequencies (MAF) ≥ 5%, observing a minimum of 56.41% in XH pigs and a maximum of 96.66% in the F_2_ generation. Considering the breeding and maintenance procedures, these results indicate that XH pigs have undergone significant inbreeding. The low-frequency variants (MAF < 5%) retained in this population were likely heterozygous sites that were incidentally preserved during inbreeding. In contrast, the F_2_ generation was primarily derived from controlled crosses between MX and LX, produced through controlled mating, exhibited a relatively simple population structure, with allele frequencies more centrally distributed around the median (Figure 2D).

### 3.3. Phylogenetic Relationships and Population Structure Analysis

**Figure 3:**
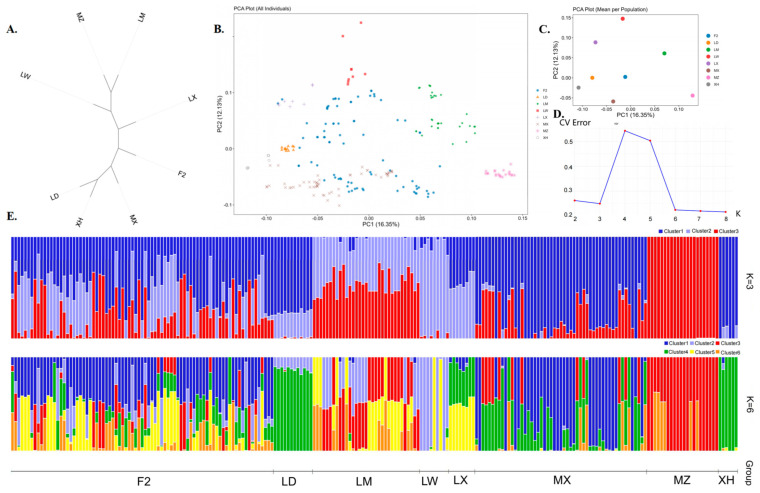
Population structure of black pigs in Xinjiang. (**A**) Phylogenetic tree between populations; (**B**) Inter-individual principal component analysis (PCA) plot; (**C**) inter-population PCA map; (**D**) CV-error line chart; (**E**) ADMIXTURE analysis (K = 3, K = 6).

A phylogenetic tree constructed using the neighbour-joining (NJ) method revealed relationships among the eight populations. In addition, a clear split was observed in the NJ tree constructed using LD pigs as the outgroup (Figure 3A). The adjacent tree showed that MZ and LW pigs were at one end of the evolutionary tree, and the genetic distance between XH and LD pigs was closest to the other end of the NJ tree, whereas the hybrid generation (MX, LX, and LM pigs) was distributed between the parents. The F_2_ generation was between the hybrid generations. The PCA results were consistent with the NJ clustering pattern described above (Figure 3B). The first two principal components explained 16.35% and 12.13% of the total variation. LD and XH pigs are clustered on the left side of the figure, LW and MZ pigs are clustered together and distributed in the upper and lower right, respectively, whereas the F1 (LM, MX, and LX pigs) and F_2_ generations are distributed across the four populations. We performed pedigree analysis using ADMIXTURE to examine admixtures across all populations. Based on our results, the optimal ancestral cluster number K was determined using a 5-fold cross-validation method. The ancestral lineage coefficient was tested for K (2–8), with K = 3 representing the optimal number of hypothetical ancestors (Figure 3C,D). Thus, it is evident that XH pigs are independent and form a unique genetic structure.

### 3.4. Genetic Kinship and Linkage Disequilibrium Patterns

**Figure 4:**
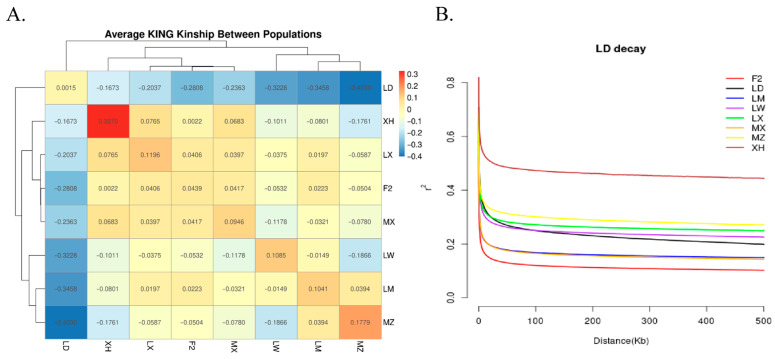
Kinship Matrix and LD Decay Analysis (**A**) Heatmap of Kinship matrix to measure the genetic similarity between different pig populations; (**B**) linkage disequilibrium decay for different pig populations.

The Kinship coefficient, calculated using PLINK2, was used to construct and visualise the average genetic kinship matrix across populations, thereby quantifying the genetic similarity (kinship strength) among different pig populations. The KING coefficient can be positive or negative, with values above diagonal lines indicating intra-population kinship and those below diagonal lines indicating inter-population kinship. For XH pigs, the estimated diagonal value was 0.32697, higher than that for their half-siblings (0.25), indicating that the XH population has undergone severe inbreeding (Figure 4A, Supplementary Table S6). Moreover, the kinship coefficients for the F_2_ generation vis-à-vis MX, LX, and LM pigs were 0.0416, 0.0400, and 0.0217, respectively, indicating that the F_2_ generation shares part of its lineage with MX, LX, and LM pigs, which is consistent with their breeding history. LD and other populations showed strong negative values, indicating strong differentiation from native Chinese pigs.

In addition, the linkage disequilibrium analysis used the r^2^ value to measure linkage disequilibrium decay, providing information on the overall diversity in each test group. Among the test populations, XH pigs exhibited the highest linkage disequilibrium levels, whereas F_2_ and LM pigs exhibited the lowest levels (Figure 4B). The results presented in the Linkage disequilibrium decay are consistent with the above θπ analysis, further indicating that XH pigs have experienced severe inbreeding and that the genetic diversity of F_2_ is effectively preserved under the existing breeding regimen.

### 3.5. Selection Signature Detection of the XH Pigs

**Figure 5:**
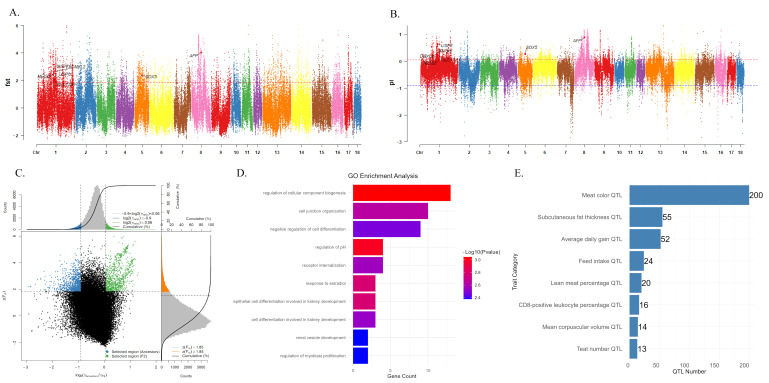
Genome-wide distribution of selection signatures detected using *F*_ST_ and θπ on 18 chromosomes from top to bottom. The *X*-axis represents 18 autosomes, and the *Y*-axis shows the statistical values for each method. The π ratio values are log2-normalised, and the *F*_ST_ values are standardised. The red line shows the 5% threshold. The blue line shows the 95% threshold. (**A**) Manhattan diagram of *F*_ST_; (**B**) Manhattan diagram of π-ratio; (**C**) distribution of dot plot of up and *F*_ST_ values (top 5% outliers), π-ratio value (top 5% outliers); (**D**) GO top 10 terms; (**E**) QTL terms and number.

To further explore the genetic improvement effect of XH pigs, in this study, we compared the genomic characteristics of parental generation with those of the F_2_ population at the population level. Population differentiation index (*F*_ST_) and nucleotide polymorphism ratio (π ratio) were used to screen the selected regions. The gene pool was established by selecting three indigenous pig breeds: XH, LW, and MZ. Subsequently, the *F*_ST_ and π ratio values between the F_2_ generation and the constructed gene pool were computed after statistical treatment (*F*_ST_ normalised by *Z*-score and the threshold set to 1.87; π ratio was log_2_ transformed, the upper and lower thresholds were 0.297 and −2.91, respectively), a total of 686 significant selected regions were identified (Figure 5A–C).

### 3.6. Genome Annotation and Functional Analyses

Utilising BEDTools (v.2.31.1), the overlapping regions were genetically annotated against the pig reference genome annotation files (Sus_scrofa.Sscrofa11.1.115.gtf; https://ftp.ensembl.org/pub/release-115/gtf/sus_scrofa/ (accepted on 20 August 2025)), resulting in the identification of a total of 406 genes (Supplementary Table S7). The GO and KEGG enrichment analysis of these genes showed that some entries, e.g., ‘ssc00760’ and ‘GO:0044087’, exhibited original *p*-values less than 0.05, but did not reach a significant level after false discovery rate correction (*p*-adjust > 0.05) (Supplementary Tables S8 and S9). Figure 5D displays the top 10 significantly enriched GO terms (*p* < 0.05). These enriched terms could be categorized into three major functional groups: kidney development-related, cell differentiation and proliferation-related, and cellular physiological processes. Considering the limited number of candidate genes, multiplex test correction may be overly conservative; therefore, we further discuss pathways related to growth and reproduction based on the existing literature and the background of the QTL traits. We downloaded 57,412 QTLs/ association records covering 410 basic traits and 1094 trait variants from PigQTLdb (v.57) (updated 26 August 2025). Notably, several QTL were significantly associated with meat color, backfat thickness, and average daily gain, indicating strong selection pressure on meat quality and growth rate during XH breeding (Figure 5E). To further narrow down the candidate gene list, we performed an intersection analysis of GO, KEGG, and QTL annotation results. The analysis showed that 20 of the candidate genes with a *p*-value of less than 0.05 overlapped with known QTLs (Supplementary Table S10), namely *SHC4*, *MAPK6*, *USP8*, *SLC12A1*, *ALB*, *SPRY1*, *SOX5*, *SLC4A4*, *GLDN*, *TLN2*, *ARPP19*, *MNS1*, *DMXL2*, *TMOD3*, *MEIS2*, *PEAK1*, *HMG20A*, *AFP*, *LMAN2*, and *NEDD4*.

## 4. Discussion

In this study, the DNBSEQ-T7 platform was used to perform high- and low-depth whole-genome resequencing of 224 individuals, including 6 XH, 22 MZ, 9 LW, 33 LM, 8 LX, 53 MX, 81 F_2_, and 12 LD pigs. Among them, 20 were subjected to 20 × high-depth sequencing (parents and excellent offspring individuals), and the rest were subjected to 2 × low-depth sequencing (Supplementary Table S1). Although this study only evaluated concordance between 20× and 1× data (87.79–89.89%), the high sequencing quality (Q30 > 93%) and existing evidence that imputation accuracy improves with coverage depth support the reliability of the 2× data actually used in downstream analyses [22,23].

In general, species under intense selection pressure exhibit lower genetic diversity, whereas indigenous and cultivated Chinese breeds exhibit higher genetic diversity, owing to traditional breeding methods involving natural or random mating [24,25]. Ecological and behavioral factors primarily influence natural mating, whereas random mating assumes equal mating opportunities among individuals. Both of them can reduce the genetic bottleneck caused by directional selection and inbreeding, thus maintaining high genetic diversity. The comparison results for He and Ho revealed intense inbreeding within the XH pig conservation population and extremely low genetic diversity. Under the existing breeding program, the genetic diversity of the F_2_ generation is effectively preserved. The calculation of the MAF also supported this view.

In addition, we explored the relationships among the sample size, He, and π values. In the absence of severe sampling bias, the He and π values did not show a trend consistent with the changes in sample size. The changes in He and π the values preliminarily indicate a positive correlation between the two. However, this relationship is not absolute, as it is influenced by factors such as population structure and mutation rate.

The NJ tree and PCA revealed that XH pigs were closer to LD pigs at one end of the evolutionary tree, whereas the LW and MZ populations were clustered at the other end, and the F_2_ generation pigs were located in the centre of the phylogenetic tree. Studies have shown that the genetic similarity between European pig breeds and those in northern China is high, and that the closer distance between XH and LD pigs may be related to their geographical location [4]. The PCA results are consistent with the clustering patterns observed in the NJ tree. The results of the mixed analysis showed that when the number of common ancestors was set to K = 3, this was the best answer, allowing MZ, LW, and XH pigs to be distinguished. This differentiation is maintained even with an increase in the number of common ancestors. At K = 6, the MZ, LW, XH, and LD pigs were clearly distinguished. To maintain genetic diversity, a core population can be established based on bloodline proportions (Figure 3E), pedigree information, and F_2_ production performance to maintain genetic diversity and support future breeding. To further preserve variation, individuals with balanced ancestry, identified via admixture analysis, were prioritized; breeding strategies were implemented to limit inbreeding accumulation, and periodic outcrossing was introduced to counteract genetic drift and gene pool contraction.

The kinship coefficient can be used to measure genetic similarity or kinship strength between different pig populations [26]. The KING coefficient can be positive or negative, with positive values indicating a close genetic relationship. Negative values suggested significant genetic differentiation or distant relationships. For XH pigs, diagonal = 0.32697, which was higher than that of the half-siblings, indicating that the XH population experienced severe inbreeding. Linkage disequilibrium was significantly more negative than in the other groups, indicating greater differentiation. The linkage disequilibrium decay attenuation graph measures the cascading imbalance in r^2^ between populations and provides information on genetic diversity. The r^2^ level of XH pigs was the highest, indicating that the chain imbalance in XH pigs was the greatest, which is consistent with the value and KING matrix conclusions.

The F_2_ generation was primarily derived from crosses between LX and MX, incorporating genetic contributions from XH, LW, and MZ lineages. In this study, these three populations (XH, LW, and MZ) were combined to form an ancestral gene pool, which was then compared with the F_2_ generation in terms of genetic structure and diversity. To verify the selection effects of the current breeding scheme on carcass meat quality, production performance, and reproductive performance, the candidate areas with high *F*_ST_ values and π ratios (top 5%) were screened. A total of 686 overlapping regions were detected within the candidate intervals, from which 406 candidate genes were identified. The pathway genes annotated to *p* < 0.05 by GO and KEGG indicate that the candidate genes are significantly involved in key biological processes, including kidney development, synaptic formation, cytoskeletal reorganization, post-translational protein modification, and inflammatory regulation, suggesting their potential roles in organogenesis, cell communication, and maintaining homeostasis. Although several GO terms showed nominal enrichment (raw *p* < 0.05), none remained significant after Benjamini–Hochberg correction (FDR > 0.05), likely due to limited sample size, a modest number of differentially expressed genes, or subtle biological effects—factors that reduce statistical power [27,28]. As the genetic improvement program for Xinjiang Black pigs advances and integrated phenotypic–genomic datasets expand, future analyses based on larger cohorts are expected to enhance detection power and enable more robust identification of functional pathways underlying key economic traits.

In order to further explore the genes related to germplasm traits, the results were intersected with the genes annotated by QTL, and 20 genes were obtained, *SHC4*, *MAPK6*, *USP8*, *SLC12A1*, *ALB*, *SPRY1*, *SOX5*, *SLC4A4*, *GLDN*, *TLN2*, *ARPP19*, *MNS1*, *DMXL2*, *TMOD3*, *MEIS2*, *PEAK1*, *HMG20A*, *AFP*, *LMAN2*, and *NEDD4*, which were associated with daily weight gain, litter size, fatty acid metabolism, and other production traits (Supplementary Table S10).

*SOX5* plays an important role in chondrocyte differentiation, and variations in its expression can lead to mild cartilage dysplasia. In porcine preadipocytes, *SOX5* promotes adipogenesis by activating the BMP-R-Smad signalling pathway. Knockdown significantly inhibited expression of the fat marker genes *PPARγ*, *aP2*, and *FAS*, and reduced lipid accumulation [29,30]. *HMG20A* was highly expressed during early porcine intramuscular fat (IMF) adipogenesis. It acts as a key negative regulator; its overexpression inhibits adipocyte differentiation in SVF and C3H10T1/2 cells, whereas its knockdown promotes adipocyte differentiation. They also play a role in cardiac development and pulsation [31,32,33]. *NEDD4* can regulate digestive function, lipid metabolism, and a series of related physiological and pathological processes; promote the repair and regeneration of damaged liver tissue; and play a protective role in fat deposition models, such as the goose liver [34,35].

*SPRY1* plays a crucial role in maintaining testicular immune homeostasis and male fertility. It is an important nodal molecule that connects inflammation, bone metabolism, and reproductive function, and is expected to be a potential therapeutic target for inflammatory bone diseases such as osteoporosis [36,37]. MNS1 is a key protein for spermatogenesis and ciliary function. It is expressed in testicular germ cells and plays an irreplaceable role in the structural integrity and function of the sperm flagella and motor cilia [38,39,40,41]. *DMXL2* (Rabconnectin-3α) is localised in GnRH neurones in the hypothalamus. It drives gonadotropin-releasing hormone (GnRH) pulse secretion by regulating vesicle exocytosis, phagocytic activity of gonadal supporting cells, and maintenance of the microenvironment. The knockout can lead to delayed puberty and hypogonadism. Additionally, it regulates insulin secretion in the pancreas and maintains blood glucose homeostasis [42,43,44]. *ALB* is closely related to sperm quality and function and regulates intracellular redox homeostasis. A study on the semen of two-flowered boars reported that ALB can be used as a novel marker of freezing adaptability [45].

*ARPP19* is crucial for mitosis (M phase) in mammals, is involved in the regulation of oocyte maturation after PKA phosphorylation, and plays a central role in the cell cycle and reproductive development [46,47,48]. *TLN2* encodes the cytoskeletal protein talin2, which maintains cell structure, adhesion, and migration and plays a critical role in embryonic development and tissue repair. This gene is expressed in chondrocytes, osteoblasts, and adipocytes, and has been reported to be associated with the regulation of the oestrous cycle and hair growth in yaks and Liaoning cashmere goats [49,50,51,52]. *SLC4A4* and *SLC12A1* (12 members of the solute carrier family 1) play key roles in ion transport and osmoregulation. A recessive missense mutation in *SLC12A1* (g.62382825G>A) is associated with hydroallantoid hydra in the foetuses of Japanese Black cattle, often leading to pregnancy failure and foetal death [53,54,55,56,57].

## 5. Conclusions

The genetic diversity and population structure of the XH population were analysed using low-depth sequencing, and the results reveal that the XH population exhibited low genetic diversity and that the genetic distance between XH and LD pigs was close. XH, LW, and MZ pigs were used as ancestral gene pools to detect selection signals in the F_2_ generation. By combining GO, KEGG enrichment analysis, and QTL annotation results, potential genetic traits related to growth, development, and reproductive ability were identified. In summary, these findings provide a better understanding of the genetics of XH pigs and provide data to support their protection and improvement.

## Data Availability

The original contributions presented in this study are included in the article/Supplementary Materials. Further inquiries can be directed to the corresponding author.

## Supplementary Materials

The following supporting information can be downloaded at https://www.mdpi.com/article/10.3390/genes17030293/s1. Table S1. The information of the samples used in this study. Table S2. Concordance between ~1× and ~20× sequencing data within the same individuals. Table S3. Count of SNP Effects. Table S4. Single nucleotide polymorphism density of each chromosome. Table S5. Population Diversity. Table S6. The average genetic kinship matrix of differrent population. Table S7. Gene Annotation of Candidate Regions. Table S8. The result of KEGG Enrichment Analyses. Table S9. The Results of Gene Ontology Enrichment Analyses. Table S10. QTLs Overlapped with Candidate Selected Regions.

## Abbreviations

The following abbreviations are used in this manuscript:

GO	Gene Ontology
He	Expected heterozygosity
Ho	Observed heterozygosity
KEGG	Kyoto Encyclopedia of Genes and Genomes
LD	Landrace
LM	Laimin
LW	Laiwu
LX	Laixin
MAF	Minor allele frequency
MX	Minxin
MZ	Min
QTL	Quantitative trait loci
XH	Xinjiang Black
